# The Toxicity of Coated Silver Nanoparticles and Their Stabilizers towards *Paracentrotus lividus* Sea Urchin Embryos

**DOI:** 10.3390/nano12224003

**Published:** 2022-11-14

**Authors:** Natalia Abramenko, Marina Semenova, Alexander Khina, Pavel Zherebin, Yurii Krutyakov, Evgeny Krysanov, Leonid Kustov

**Affiliations:** 1 N.D. Zelinsky Institute of Organic Chemistry RAS, 47 Leninsky Prospect, Moscow 119991, Russia; 2 A.N. Severtsov Institute of Problems of Ecology and Evolution RAS, 33 Leninsky Prospect, Moscow 119071, Russia; 3 N.K. Koltzov Institute of Developmental Biology RAS, 26 Vavilov Street, Moscow 119334, Russia; 4 Department of Chemistry, Lomonosov Moscow State University, 1-3 Leninskie Gory, Moscow 119991, Russia; 5 National Research Center “Kurchatov Institute”, 1 Kurchatov Square, Moscow 123182, Russia

**Keywords:** silver nanoparticles, ecotoxicity, fate, sea urchin embryo, stabilizers, marine species

## Abstract

Surface modification of nanoparticles with different stabilizers is one of the most widely used methods to improve their stability and applicability. Silver nanoparticle (AgNPs) dispersions with biologically active stabilizers have great potential as plant protection products with synergetic antimicrobial properties and sufficient stability in terms of field application. The obtained AgNPs dispersions have the ability to enhance growth, increase yield and give better protection to various crops. At the same time, it is important to determine the fate, stability, and ecotoxicity of the applied nanosized products. The toxic effects of AgNPs dispersions and their constituents, organic stabilizers and additives, were evaluated using a phenotypic sea urchin embryo assay. Certain AgNPs dispersions with organic stabilizers demonstrated sufficient stability, even in seawater. The toxicity of the AgNPs decreased with the increasing tendency to agglomerate in seawater. Furthermore, the applied stabilizers were hazardous towards sea urchin embryos. They caused pronounced embryo abnormalities at 0.25–2.6 mg/L concentrations. AgNPs exhibited a lethal effect at concentrations that were equal to the MLC or exceeded the MEC of their stabilizers. Silver ions were more toxic towards sea urchin embryos than AgNPs.

## 1. Introduction

Silver nanoparticles (AgNPs) are widely applied in medicine, industry, agriculture due to their antimicrobial properties [1,2]. However, the tendency of AgNPs to aggregate and precipitate can limit their use. The main disadvantage of products based on AgNPs and their derivatives is the insufficient aggregative stability of silver dispersions in the presence of electrolytes. Thus, there is a need for enhancement of the effectiveness of nano-formulated products; namely, to increase the stability of such dispersions in aqueous environments in the presence of inorganic ions. To prevent aggregation, AgNPs dispersions should contain additives and stabilizers, such as citrate, polymers, and surfactants. Generally, citrate-stabilized AgNPs are insufficiently stable in an aqueous environment [3]. On the contrary, the addition of anionic and cationic organic polymers and surfactants, as well as their combinations, can significantly increase the aggregative stability of AgNPs dispersions in the presence of electrolytes [4]. In addition, some polymer and surfactant stabilizers of AgNPs exhibit their own antimicrobial properties [5]. Therefore, such stabilizers could be applied to obtain AgNPs featuring sufficient aggregative stability, enhanced antimicrobial efficiency and desirable plant protection properties [4,6]. The fungicidal properties of AgNPs, as well as their ability to serve as plant immune system elicitors, make it possible to apply AgNPs in commercial plant protection products and pesticides [6,7,8].

Wide practical application of AgNPs increases the probability of their release into the environment. Thus it is critical to evaluate their environmental fate and ecotoxicity. It was reported that AgNPs dispersions obtained with different organic stabilizers exhibited toxicity against *Saccharomyces cerevisiae* cells [8] and *Danio rerio* embryos [9]. Clearly, the fate and behavior of AgNPs in fresh and seawater media is anticipated to be different, same as their activity/toxicity with respect to different species. To evaluate the toxicity of AgNPs dispersions towards marine species, the effects of AgNPs, their additives, and stabilizers were examined using a sea urchin embryo model. Sea urchin embryos are well-studied marine organisms inhabiting all oceans across the world. They are considered to be a marine species sensitive to different toxicants in seawater [10,11]. Sea urchin embryos have several benefits as an animal model in ecotoxicology, such as simple production of a large number of gametes, facile and cheap embryo rearing, and a relatively large size and optical transparency that facilitate monitoring in situ with low magnification microscopy [12]. The effects of AgNPs on sea urchin embryos have been studied previously [13,14,15]. It was shown that AgNPs may affect the sea urchin species and induce different processes, resulting in a delay in development, fertilization failure, and skeletal deformations [13,14,15]. However, the tested AgNPs were stabilized using citrate, glucose, or poly(allylamine) and demonstrated poor stability in seawater. Thus, the observed toxicity was caused by dissolution of silver ions of the surface of AgNPs.

In this article, the, dispersions of synthesized AgNPs, as well as commercial AgNPs samples, were studied in seawater. The aggregative stability and behavior of AgNPs dispersions were analyzed the in marine aquatic system. A comparative study of the toxic effects of selected stable AgNPs dispersions, stabilizers, additives, and Ag^+^ ions was conducted, emphasizing the evaluation of the relative impact of stabilizers on the overall AgNPs toxicity and fate.

The present study extends this area of research to stable AgNPs, gaining knowledge of the comparative effects of AgNPs dispersions, stabilizers, additives, and the Ag^+^ ion exposure of sea urchin embryos to AgNPs. This research is specifically focused on how AgNPs dispersions and their components, such as stabilizers and additives, affect the embryogenesis of sea urchin, in terms of developmental disorders and malformations in the embryo.

## 2. Materials and Methods

### 2.1. Reagents and Materials

Silver nitrate (99.9%, Sigma-Aldrich, St. Louis, MO, USA), sodium borohydride (VenPure, 99%, Acros Organics, Morris Plains, NJ, USA), sodium nitrate (99%, Sigma-Aldrich, St. Louis, MO, USA), sodium tetraborate decahydrate (99%, Sigma-Aldrich, St. Louis, MO, USA), sodium lauryl ether sulfate (SLES, 70% aqueous solution, Hansa, Cologne, Germany), sodium coco aminodipropionate (AMA, 38% aqueous solution, Lakeland Laboratories Ltd., Manchester, UK), sodium tallow amphopolycarboxyglycinate (STAPCG, 30% aqueous solution, additionally containing up to 10% NaCl, Akzo Nobel, Amsterdam, The Netherlands), sodium alkyldiaminoethyl glycinate (SADG, 30% aqueous solution, FUJIFILM Wako Corporation, Guangzhou, China), polyhexamethylene biguanide hydrochloride (PHMB, 20% aqueous solution, Arch Chemicals, Norwalk, CT, USA), and red sea salt (powder, Red Sea, Israel) were used in the experiments. AgNPs were synthesized by chemical reduction using procedures developed previously [5,16]. All aqueous solutions were prepared with double-distilled water.

### 2.2. Characterization of AgNPs

Microphotographs of AgNPs were obtained using an electron microscope (Leo 912 AB Omega, Carl Zeiss, Oberkochen, Germany) with an operating accelerating potential of 100 kV. Samples were prepared by spreading 1–2 µL of the diluted AgNPs dispersion onto copper grids coated with Formvar^TM^ (d = 3.05 mm), which were then dried in the open air. The average initial diameter of AgNPs was obtained from TEM images using ImageJ 1.53 software (National Institute of Mental Health, Bethesda, MD, USA). The elemental structure was identified using X-ray diffraction analysis (“dark field” method). The non-covalent stabilization mechanism of AgNPs and the presence of stabilizer molecules on the NPs surface were verified using the XPS method.

The XPS study in this research was carried out using a LAS-3000 (Riber, Bezons, France) spectrometer equipped with a hemispherical ESCA electron analyzer OPX-150. A monochromatic Al Kα X-ray source (1486.6 eV) was used as the source of exciting radiation. A working pressure of 10^−10^ was maintained.

The measurements of UV–vis absorption spectra were performed in quartz cuvettes using a Varian Cary 100 spectrophotometer (Varian, Inc., Palo Alto, CA, USA). UV-vis spectroscopy was also used to monitor the changes in the colloidal stability of tested samples in artificial seawater (38‰ salinity, pH 8.2–8.4) and distilled water at room temperature. The optical absorption spectra of the AgNPs dispersions (10–15 mg/L) in distilled water and artificial seawater were recorded using quartz cuvettes with a path length of 10 mm. Sufficient reduction in the intensity of the characteristic absorption bands of AgNPs at 400–420 nm for the tested dispersion was considered as the onset of coagulation.

In addition to TEM measurements, the hydrodynamic size and ζ-potential of the AgNPs dispersions were determined using dynamic light scattering (DLS) [17]. The absolute values of the ζ-potential were used as the criterion for the stability of the colloidal dispersions. AgNPs with the absolute value of the ζ-potential greater than 20 and 30 mV are considered moderately and highly stable, respectively. Measurements were performed using a Delsa^TM^Nano C particle analyzer (Beckman Coulter, Q2 Beckman Coulter, Brea, CA, USA) at a wavelength of 658 nm using the Delsa Nano Software package.

### 2.3. Phenotypic Sea Urchin Embryo Assay

Sea urchin embryo tests were conducted according to a previously reported protocol [18]. Adult sea urchins, *Paracentrotus lividus* (*P. lividus*), were collected from the Mediterranean Sea off the Cyprus coast and kept in an aerated seawater tank. Gametes were obtained by intracoelomic injection of an aqueous 0.5 M KCl solution. Eggs were washed with filtered seawater and fertilized by adding drops of diluted sperm. Embryos were cultured at room temperature under gentle agitation with a motor-driven plastic paddle (60 rpm) in filtered seawater at a concentration of 400–2200 eggs (embryos)/mL. This interval was considered optimal, both for microscopic observation, and for obtaining an even monolayer in six-well plates that was suitable for reproducible and tractable tests. For treatment with the test compounds, 5 mL aliquots of the egg/embryo suspension were transferred to six-well plates, which offered a favorable topology/volume (5 mL). The embryos were incubated as a monolayer at a concentration of 400–2200 embryos/mL. In each experiment, the embryo concentration in all wells was the same. The embryos were incubated at room temperature (20–26 °C) in filtered seawater with pH values of 7.65–8.22, depending on the seasonal conditions. According to long-term observations, these intervals are beneficial for successful embryo development. The embryos were observed with a Biolam light microscope (LOMO, St. Petersburg, Russia). Sea urchin embryo development was monitored until the four-arm mid-pluteus stage, 34–36 h post-fertilization. Effects of AgNPs, additives, and stabilizers were assessed by exposing fertilized eggs (FE, 8–19 min post-fertilization) and hatched blastulas (HB, 8–10.5 h post-fertilization) to 2-fold decreasing concentrations of the tested samples. The effects were estimated quantitatively, at the minimum effective (threshold) concentration that induced developmental abnormalities or mortality (lethal effect), and at 2-fold lower concentration when the tested samples failed to produce these effects. Developmental abnormalities were detected at the cleavage (2.5 and 5.5 h post-fertilization), hatching (9 h post-fertilization), mesenchyme blastula stage (2 h after hatching, 10–12 h post-fertilization), prism, early, and mid-pluteus stages. Microphotographs were obtained using an AmScope binocular microscope with an MU500 digital camera (United Scopes LLC, Irvine, CA, USA). The embryos were immobilized with 5-[(6,7-dimethoxy-1,3-benzodioxol-5-yl)methyl]-3-(4-methoxyphenyl)-4,5-hydroisoxazole at a concentration of 2 μM for 20 min [19]. Experiments with the sea urchin embryos fulfilled the requirements of biological ethics. Artificial spawning does not cause animal death, embryos develop outside the female organism, and both post-spawned adult sea urchins and the excess of intact embryos are returned to the sea, their natural habitat.

Statistical processing of results was carried out using the program GraphPad Prism version 8.0 (GraphPad Software, San Diego, CA, USA).

## 3. Results and Discussion

### 3.1. Characterization of Nanosilver Samples

**Figure 1:**
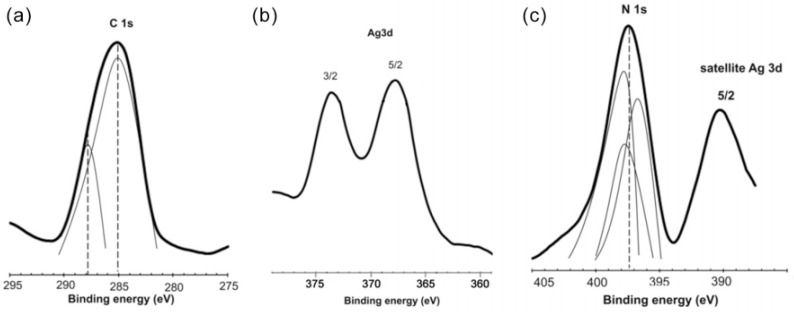
Typical XPS spectra for the Ag/PHMB sample (**a**) C 1s, (**b**) Ag 3d, and (**c**) N 1s photoelectron spectra (counts per second, cps vs. binding energy, B.E.).

The non-covalent stabilization mechanism of AgNPs and the presence of stabilizer molecules on the NPs surface were verified using the XPS method (Figure 1).

The Ag 3d spectrum exhibits peaks at 368.3 eV (3d_5/2_) and 374.0 eV (3d_3/2_), which correspond to silver in the *zero*-valence state. The peak with the highest intensity (binding energy 285.1 eV) may correspond to aliphatic carbon. The peak with a lower intensity (binding energy 288.0 eV) corresponds to imine carbon. The presence of a signal in the binding energy range 400–405 eV corresponds to the presence of nitrogen atoms. Imine nitrogen in the PHMB chain can effectively stabilize AgNPs, by formation of a strong coordination bond, Ag-N.

**Figure 2:**
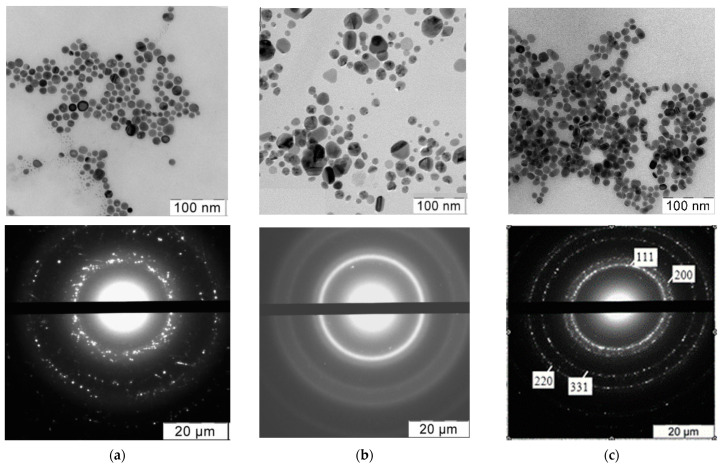
Typical electron microphotographs and X-ray diffraction patterns of (**a**) Ag/PHMB; (**b**) Ag/STAPCG; (**c**) Ag/SLES nanoparticles. Microphotographs and diffractograms of other nanoparticles were similar.

The shape and size of the synthesized AgNPs were determined using TEM (Table 1). Figure 2 shows typical TEM pictures and reflections of the Ag nanocrystals. All AgNPs had an almost ideal spherical shape, and their average diameter was in the range of 5–20 nm.

The average hydrodynamic diameter and the ζ-potential of AgNPs were measured using DLS, and the results are summarized in Table 1.

Various physicochemical processes, such as dissolution and agglomeration, surface modification and chemical transformation, are crucial factors that influence AgNPs toxicity. For instance, the aggregation of NPs can dramatically reduce the active surface area of silver particles and, as a consequence, their influence on sea urchin embryo development. Therefore, it was important to estimate the stability of AgNPs dispersions for further biological evaluation, i.e., for an adequate assessment of their effect on the model organism. Thus, the colloidal behavior of the AgNPs in distilled water and artificial seawater during 24 h (equal to the average period of exposure of sea urchin embryos to AgNPs) was studied initially.

### 3.2. Environmental Fate

**Figure 3:**
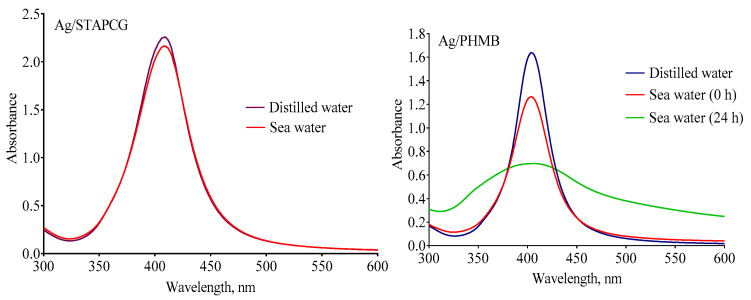
Examples of UV-vis spectra for stable (Ag/STAPCG) and unstable (Ag/PHMB) AgNPs diluted in distilled water and seawater (10–15 mg/L).

There is a certain possibility of AgNPs being released into natural aquatic systems. In natural waters, AgNPs can accumulate, sediment, dissolve and react with outer ions and natural organic materials. All these processes influence the fate and ecotoxicity of AgNPs [20,21]. Thus, it is critical to evaluate the behavior of AgNPs in natural waters. It is known that salinity plays a very important role in the fate of AgNPs. For instance, it was shown in [22] that citrate-coated AgNPs remained stable in low-salinity water (up to 0.08 mg/L of dissolved salts). With increased salinity, destabilization and sedimentation of AgNPs occurs due the higher ionic strength caused by free ions such as Cl^−^, SO_4_^2−^, or S^2−^. Furthermore, it should be noted that citrate-stabilized [13,14] and poly(allylamine)-coated AgNPs [15], as well as commercial Polytech AgNPs [23] were reported to form aggregates in seawater. Therefore, the stability of AgNPs dispersions in distilled water and seawater was examined using UV-vis spectroscopy (Figure 3).

A wide band with a maximum near 400–420 nm is associated with the plasmon absorption of AgNPs. Thus, a decrease in the intensity of the band corresponds to the loss of the nanosize of the silver particles, caused by aggregation. According to UV-vis spectroscopy, all samples were stable in distilled water. In terms of the seawater (38 ‰ salinity, pH 8.2–8.4, room temperature), the decrease in the intensity of plasmon absorption was no more than 5% for Ag/STAPCG, Ag/PHMB&SLES, Ag/SADG, and Ag/AMA within the exposure period of the sea urchin embryos. Thus, the Ag/STAPCG, Ag/PHMB&SLES, Ag/SADG, and Ag/AMA dispersions could be considered stable during the experiment. In contrast, a dramatic reduction of the plasmon absorbance intensity was observed for the Ag/PHMB and Ag/SLES samples. Moreover, Ag/PHMB and Ag/SLES seawater dispersions became completely colorless within one day of storage, indicating the loss of their colloidal stability, whereas other samples preserved their color, which demonstrated sufficient stability.

The hydrodynamic diameter (D) measured by DLS can also serve as an indicator of the colloidal stability of the particles (Table 1). Table 1 summarizes the AgNPs characteristics in distilled water and seawater.

According to the data summarized in Table 1, Ag/PHMB and Ag/SLES lose their colloidal stability in seawater, as their hydrodynamic diameter increases significantly in these conditions, which shows the formation of aggregates. Inversely, Ag/AMA dispersion remained stable in the seawater, despite the change in its hydrodynamic diameter, which also indicated the formation of aggregates up to 200 nm in size.

The dispersions of AgNPs stabilized using STAPCG, SADG, and AMA were stable in seawater. Notably, these stabilizers have a similar chemical structure. Namely, they are all anionic surfactants, and have one or more carboxyl head groups and one or more nitrogen atoms. On the contrary, AgNPs dispersions stabilized using an anionic surfactant with a sulfate group (SLES) or cationic polymer (PHMB) turned out to be unstable in seawater. Thus, the stability of AgNPs dispersions in seawater depends on the chemical structure of the stabilizing agents, and anionic surfactants containing nitrogen and carboxyl groups are optimal in this regard.

In addition, AgNPs simultaneously stabilized with SLES and PHMB had the increased aggregative stability in seawater, which presumably was due to the formation of a complex coating of these two stabilizers on the surface of the AgNPs, which created both electrostatic and steric protection.

AgNPs produced by different methods have unique characteristics and properties. Accordingly, their behavior in aquatic media and activity/toxicity is anticipated to be different. Therefore, the study of distinct AgNPs ecotoxicity against aquatic/marine organisms is of particular importance.

### 3.3. Toxicity of AgNPs

Considering the reported hazardous effect of AgNPs towards marine organisms [13,14,15,23,24,25], their potential accumulation in nature, as well as their increasing commercial use [13,14], we investigated the toxicity of AgNPs in a phenotypic sea urchin embryo assay [18]. Fertilized eggs (FE) or hatched blastulas (HB) were exposed to a stabilizer or AgNPs. The subsequent embryo development was monitored in the presence of the tested samples until the four-arm pluteus stage (the beginning of active feeding (32–36 h post-fertilization) or until embryo death. Developmental abnormalities, such as cleavage alteration/arrest, inhibition of hatching, gastrulation, and spiculogenesis, were detected at cleavage (2.5 and 5.5 h post-fertilization), hatching (9 h post-fertilization), mesenchyme blastula stage (2 h after hatching, 10–12 h post-fertilization), and prism, early, and mid-pluteus stages. Embryo disintegration or malformation, together with immobilization at the bottom of the vessel, was considered a lethal effect. The minimum effective concentration that caused developmental abnormalities (MEC), as well as the minimal lethal concentration (MLC), were determined for FE and HB treatments. In all experimental conditions, duplicate measurements showed no differences in MLC and MEC values.

**Figure 4:**
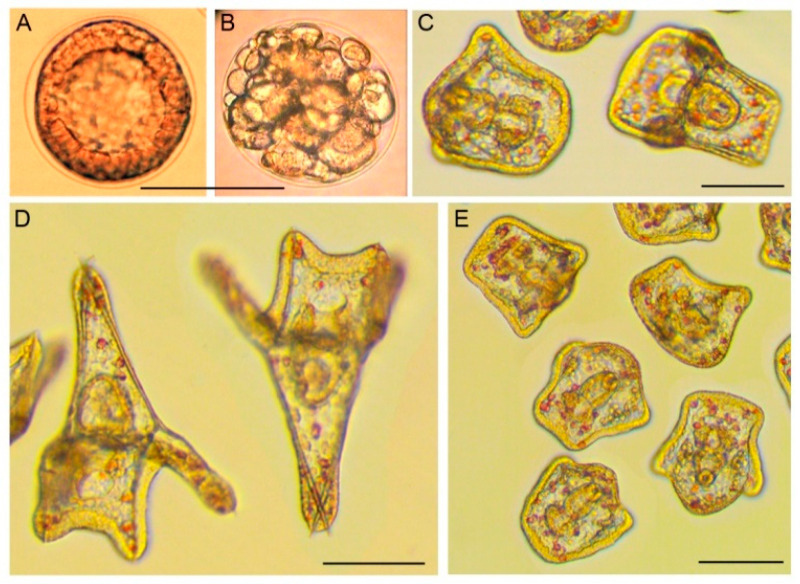
Typical effects of AgNPs and stabilizers on sea urchin embryos, as exemplified by Ag/AMA (**B**,**E**) and AMA (**C**). Control: (**A**) intact blastula, 5.5 h post-fertilization; (**D**) intact pluteus, 34 h post-fertilization. (**B**) Cleavage disorder caused by Ag/AMA at 1.0/8.0 mg/L exposed to fertilized eggs, 5.5 h post-fertilization. The subsequent embryo death was observed. (**C**) Viable motile retarded plutei (34 h post-fertilization) formed after hatching blastulae treatment by AMA at 4 mg/L, 25 h of exposure, 34 h post-fertilization. (**E**) Viable motile small-size early plutei without skeletal spiculae or with thin short spiculae formed after hatching blastulae treatment by Ag/AMA at 0.25/2 mg/L, 25 h of exposure, 34 h post-fertilization. The embryos in C–E were immobilized with 5-[(6,7-dimethoxy-1,3-benzodioxol-5-yl)methyl]-3-(4-methoxyphenyl)-4,5-hydroisoxazole at a concentration of 2 μM for 20 min [19]. Incubation temperature: 23 °C. Scale bars: 100 µm.

AgNPs and stabilizers were found to exhibit pronounced effects on *P. lividus* embryos, causing different developmental disorders that could culminate in embryo death. Typical effects of AgNPs on sea urchin embryos are shown in Figure 4. First, the maximal tolerated (or: No Observed Effect Concentration, NOEC) concentrations of the stabilizer (or stabilizing mixture used in AgNPs dispersions preparation) were determined. Next, to identify the toxicity of the AgNPs themselves, avoiding the possible toxic impact of the stabilizer, AgNPs were tested at the maximal concentration that did not exceed the NOEC of the stabilizer contained in the AgNPs. The effects of the stabilizers are presented in Table 2.

Based on the EPA guidelines [26], most of the substances showed moderate or low toxicity. PHMB failed to cause embryo mortality up to the 5 mg/L concentration. Fertilized eggs were more sensitive to stabilizers compared to the blastula after hatching. MEC values for the majority of the stabilizers were similar, except for those of stabilizer mixtures, whereas MLC values exhibited some variability. In mixtures of substances, the toxicity of the system was attributed to the more toxic component. To compare the effect of each component in the mixture, we expressed toxicity data as separate effects of each component. In comparison with the data of the single stabilizers (SLES and PHMB), the MEC data for the mixtures were practically equal to the SLES toxicity.

Next, we carried out experiments with AgNPs dispersions taking into account the safe concentration (NOEC) of the stabilizing agents. AgNPs dispersions samples were screened for toxicity at double-decreased concentrations, starting from the maximal tolerated concentration of the stabilizer (or the stabilizing mixture) in the AgNPs dispersions sample. The effect of Ag^+^ ions was examined using silver nitrate salt. All tested AgNPs were less toxic than Ag^+^ ions. A summary of the data for the tested AgNPs and Ag^+^ ions is shown in Table 3.

Some of dispersions were excluded due to their instability in aqueous media and precipitation during the experiments. For instance, Ag/PHMB formed aggregates in seawater, which were clearly visible under low magnification at a concentration of AgNPs ≥ 0.3 mg/L. Therefore, it was impossible to estimate the exact AgNPs concentration in the tested solution. Nevertheless, most of the AgNPs colloids remained stable during the study (up to 34–36 h) according to the UV-vis spectrophotometry and DLS data, and there were no signs of AgNPs precipitation in the test beakers.

Some stabilizers exhibited high toxicity towards *P. lividus* embryos, and the toxic effect of AgNPs dispersion was mainly attributed to the toxicity of the stabilizer. For example, in the case of the Ag/STAPCG and Ag/PHMB&SLES samples, the toxicity can be explained by the presence of the stabilizers STAPCG and PHMB&SLES as the most toxic components. Considering the location of SLES in the outer layer of the AgNPs structure and the low toxicity of PHMB towards sea urchin embryos, we suggested that the toxic effect of Ag/PHMB&SLES was indeed equal to the toxicity of SLES.

Generally, FE were more sensitive to stabilizers and AgNPs than HB, so that MLC FE < MLC HB for most of the tested samples. The observed lethal effect correlated with the duration of exposure and was observed after 2–26 h of treatment, depending on the sample concentration and the developmental stage (Table S1, Supporting Information). Usually, the lethal effect of stabilizers on the hatched blastula stage developed more quickly, as evidenced by a lower duration of treatment resulting in embryo death.

According to the literature, poly(allylamine)-coated, citrate- and β-D-glucose-stabilized AgNPs caused various developmental abnormalities in sea urchin embryos. Namely, cleavage alteration and arrest, exogastrulation, and aberrant skeletal morphology were detected after pre-fertilization sperm treatment using AgNPs with the size of 1–10 nm at a 0.0001–1 mg/L concentration, suggesting that AgNPs could somehow be transferred from male gametes with further accumulation and retention in eggs and early embryos [23,27]. The embryotoxicity of AgNPs was manifested in developmental retardation and arrest, accompanied by inhibition of larval skeletogenesis [13,14,25]. A similar toxic effect was identified for β-d-glucose-stabilized AgNPs at a concentration of 0.3 × 10^13^ AgNPs/mL [28]. Agglomerates of citrate-stabilized AgNPs were observed within the larva at the four-arm pluteus stage, after exposure to 0.3 mg/L AgNPs at the four-cell stage [25]. Citrate-stabilized AgNPs at 0.03–3 mg/L severely disrupted development of *P. lividus* embryos, when added to fertilized eggs, four-cell stage, blastula, and gastrula. Toxic effects of AgNPs of a different size and structure toward various sea urchin species at different developmental stages were reported [24]. Remarkably, most of studied AgNPs were characterized as poorly stable in seawater. As a consequence, their toxicity could be attributed to silver ions dissociated from the AgNPs surface.

It is known that NPs undergo various transformations under natural conditions, such as aggregation, oxidation, disintegration, interaction with natural organic materials, etc. [29]. All these processes influence the NP activity, and their effects should be given consideration. The physical and chemical properties of AgNPs, namely, the size, tendency for agglomeration, shape, surface chemistry, and charge, were suggested as the most important factors responsible for the unique activity and toxicity of NPs in comparison with their bulk counterparts [30,31]. The tested AgNPs had different mean diameters and charges, and demonstrated different stabilitiy in the tested media. In our experiment, we did not observe significant size-dependent toxicity of AgNPs. The samples Ag/STAPCG and Ag/SADG, with a similar hydrodynamic diameter in seawater (D) of 75.22 nm and 70.73 nm, respectively, caused different toxic effects on sea urchin embryos (for Ag/STAPCG and Ag/SADG, the MEC values were 0.06 and 0.125 mg/L, respectively). On the contrary, Ag/SADG, Ag/AMA, and Ag/SLES, with a different particle size (D = 70.73, 200.3, and 299.3 nm in seawater, respectively) displayed similar toxicitiy towards *P. lividus* embryos (MEC FE = 0.120–0.125 mg/L). We supposed that the toxicity of the AgNPs correlated with their complex physicochemical properties and could not be explained only by the size.

In this regard, the correlation between AgNPs embryotoxicity and their charge and stability in the tested media could be assumed. According to the particle characterization in the tested media, most of the negatively charged AgNPs were stable during the exposure. The ζ-potential of the Ag/STAPCG and Ag/PHMB&SLES samples increased in seawater, suggesting sufficient AgNPs stability in the experimental conditions. Interestingly, these samples were more hazardous towards *P. lividus* embryos. In contrast, less stable AgNPs with a tendency for aggregation and sedimentation were less toxic towards sea urchin embryos. For instance, positively-charged Ag/PHMB NPs were less stable, and the Ag/PHMB sample did not display significant toxicity in comparison with the other samples. On the contrary, positively-charged AgNPs usually show higher antimicrobial activity, due to electrostatic attraction to negatively-charged bacterial cells [32].

The charge, size, and tendency of AgNPs to aggregate depend directly on the synthesis conditions and the nature of the stabilizer. Nevertheless, we could not ignore the toxic effect of the additives. Thus, the effects of other possible components of AgNPs dispersions on the sea urchin embryos were tested. No effects were observed for the mixture of NaNO_3_/Na_2_B_4_O_7_ up to final concentration of 22 mg/L/56 mg/L, which corresponded to 30 mg/L of Ag in AgNPs. The results confirmed the lack of toxicity of the salts used in the synthesis of AgNPs towards *P. lividus* embryos. In contrast, several stabilizers were highly toxic to *P. lividus* embryos, such as SLES and STAPCG. The threshold lethal concentration values, MLC, for the dispersions of Ag/SLES and Ag/STAPCG were equal to the MLCs of the respective stabilizers.

AgNPs effects on sea urchin embryos were previously examined. However, all the AgNPs studied before were stabilized using citrate, glucose, or poly(allylamine), and were characterized as weakly stable in seawater. As a consequence, their toxicity could be attributed to silver ions dissociated from the AgNPs surface.

In contrast, we studied AgNPs stabilized by several organic surfactants, and some of the AgNPs showed pronounced stability in seawater. The applied stabilizers are substances widely used in cosmetics, chemical industry, and agriculture. Their combination with AgNPs helps create dispersions with potential for practical application. The fate of these AgNPs in seawater and their toxicity towards marine species have not been studied previously. AgNPs in combination with antimicrobial stabilizers have enriched antimicrobial properties and sufficient stability, even in seawater. This study of the aggregative stability of AgNPs with different stabilizers can expand the area of their application. On the one hand, it is practically useful to apply concentrated NPs dispersions that can be diluted with natural waters. On the other hand, AgNPs with improved stability can be used in a mixture with other pesticides or fertilizers.

This comparative toxicity study of AgNPs dispersions and individual stabilizers can improve the understanding of the reasons behind the toxicity of coated AgNPs dispersions. The main novelty is the high toxicity of the stabilizers observed using the sea urchin embryo model. This toxicity was so significant that it “camouflaged” the toxic effect of the AgNPs themselves. It was shown that the chemical nature of an organic stabilizer has an impact on the overall biological effect of the AgNPs dispersions.

**Figure 5:**
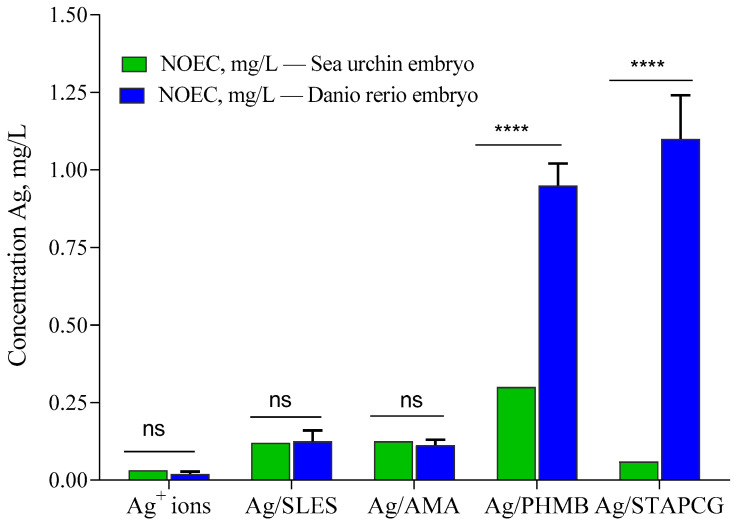
Effects of AgNPs on sea urchin *P. lividus* and zebrafish *Danio rerio* embryos. Sea urchin embryos were exposed to AgNPs at the fertilized egg (FE) stage. NOEC: maximal no observed effect concentration (****—statistically significant; ns—statistically not significant).

In summary, the obtained results demonstrate that AgNPs, as well as stabilizing agents, exhibit a toxic effect towards sea urchin *P. lividus* embryos. However, the toxicity of AgNPs was significantly lower than that of Ag^+^ ions. Similar results were obtained during our previous work, where zebrafish *Danio rerio* embryos were used as a test model (Figure 5) [9].

Both species were more sensitive to Ag^+^ ions than to AgNPs dispersions. Noticeably, sea urchin embryos demonstrated higher sensitivity towards most of the tested samples in comparison with *Danio rerio* embryos. Similarly to the previous study [8,9], the toxicity of Ag/AMA was found to be attributed to the AgNPs themselves, not to the stabilizer. On the other hand, several stabilizers, such as SLES, PHMB, and, to a lesser extent, STAPCG, demonstrated pronounced toxic effects towards aquatic species. Our study provided evidence that different environmental conditions could have a dramatic impact on the AgNPs colloidal stability, which leads to significant reduction of their toxicity towards animal species.

## 4. Conclusions

Combination of AgNPs dispersions with biologically active stabilizers may expand their applicability as such systems have improved antimicrobial properties and sufficient stability. However, when using such stabilizers, their impact on the environment must be taken into account. Biological assessment of the AgNPs toxicity in a sea urchin embryo model revealed that both stabilizers and AgNPs impaired embryonic development. AgNPs exhibited a lethal effect at concentrations that were equal to the MLC, or exceeded MEC, of the respective stabilizers contained in the AgNPs dispersions, while Ag^+^ ions were more toxic than AgNPs. Thus, the chemical nature of the stabilizer may play a crucial role in the stability and overall biological effect of AgNPs dispersions. Taking into account the relatively low concentration of AgNPs dispersions effectively used in agriculture, they can significantly increase yield and reduce the spread of crop diseases, with less negative impact on the environment in comparison with traditional plant protection products.

## Figures and Tables

**Table 1:** Composition and physicochemical characteristics of the AgNPs used in the study.

Sample Ag/Stabilizer	C_Ag/Stabilizer_, mg/L	D (TEM), nm	D, nm ^a^		ζ-Potential, mV ^b^	
			Distilled Water	Seawater	Distilled Water	Seawater
Ag/STAPCG	3000/48,000	19.4 ± 9.4	52.5 ± 5.9	75.2 ± 13.1	−52.9	−75.9
Ag/PHMB&SLES	500/500/5200	14.9 ± 10.2	59.5 ± 3.1	33.4 ± 9.9	−54.7	−61.5
Ag/SADG	500/2000	12.8 ± 7.1	61.9 ± 3.8	70.7 ± 10.8	−36.1	−22.4
Ag/AMA	500/4000	8.0 ± 1.9	52.2 ± 9.6	200.3 ± 2.9	−50.5	−24.4
Ag/PHMB	500/500	6.6 ± 4.8	48.8 ± 0.5	1139.0 ± 57.8	42.8	NA ^c^
Ag/SLES	100/1000	7.5 ± 3.6	45.8 ± 1.5	299.3 ± 20.5	−46.8	NA ^c^

^a^ D: hydrodynamic diameter of AgNPs ± standard deviation by DLS (24 h). ^b^ ζ-Potential—NPs with a zeta potential higher than ± 20 mV and higher than ± 30 mV were considered moderately and highly stable, respectively. ^c^ NA: Not available due to AgNPs sedimentation.

**Table 2:** Effects of stabilizers on sea urchin embryos.

Stabilizer	MLC FE, mg/L	MLC HB, mg/L	MEC, mg/L
STAPCG	2.4	4.8	2.4
PHMB/PHMB&SLES ^a^	1	1	0.25
SLES/PHMB&SLES ^b^	10.4	10.4	2.6
SADG	4	4	2
AMA	8	20	2
PHMB	>5	>5	2.5
SLES	5	10	2.5

MLC: minimal lethal concentration. MEC: minimal effective concentration. ^a^ PHMB toxicity in the mixture. ^b^ SLES toxicity in the mixture. Duplicate measurements showed no differences in the MLC and MEC values.

**Table 3:** Effect of AgNPs on sea urchin embryos.

AgNPs	AgNPs MLC, mg/L		AgNPs MEC, mg/L	
	FE	HB	FE	HB
Ag/STAPCG	0.15 ^a^	0.30 ^a^	0.06	0.06
Ag/PHMB&SLES	0.50	1.00 ^a^	0.25 ^a^	0.25 ^a^
Ag/SADG	0.50 ^a^	0.50 ^a^	0.13	0.13
Ag/AMA	0.50	0.50	0.13	0.13
Ag/PHMB ^b^	2.50	>2.50	0.30	1.25
Ag/SLES	0.50 ^a^	0.50 ^a^	0.12	0.12
Ag ions	0.064	>0.13	0.03	0.064

^a^ AgNPs MLC/MEC were equal to the respective stabilizer MLC/MEC. ^b^ Ag/PHMB at a concentration of ≥0.30/0.30 mg/L formed crystals in seawater. Duplicate measurements showed no differences in MLC and MEC values.

## Data Availability

Not applicable.

## Supplementary Materials

The following supporting information can be downloaded at: https://www.mdpi.com/article/10.3390/nano12224003/s1, Table S1: Lethal effects of Ag NPs, stabilizers, and Ag^+^ ions on sea urchin embryos.
