# Supplementary material for: The Toxicity of Coated Silver Nanoparticles and Their Stabilizers towards Paracentrotus lividus Sea Urchin Embryos
Source: Nanomaterials (Basel). 2022 Nov 14;12(22):4003. doi: 10.3390/nano12224003 (PMC9695290; doi:10.3390/nano12224003)
Supplement: Supplementary file 1 [file nanomaterials-12-04003-s001.zip › nanomaterials-2006062-supplementary.pdf]

nanomaterials

Supplementary Materials

The Toxicity of Coated Silver Nanoparticles and Their Stabilizers towards *Paracentrotus lividus* Sea Urchin Embryos

Natalia Abramenko [1,2], Marina Semenova [3], Alexander Khina [4], Pavel Zherebin [4], Yurii Krutyakov [4,5], Evgeny Krysanov [2] and Leonid Kustov [1,4,*]

1 N.D. Zelinsky Institute of Organic Chemistry RAS, 47 Leninsky Prospect, Moscow 119991, Russia
2 A.N. Severtsov Institute of Problems of Ecology and Evolution RAS, 33 Leninsky Prospect, Moscow 119071, Russia
3 N.K. Koltzov Institute of Developmental Biology RAS, 26 Vavilov Street, Moscow 119334, Russia
4 Department of Chemistry, Lomonosov Moscow State University, 1-3 Leninskie Gory, Moscow 119991, Russia
5 National Research Center "Kurchatov Institute", 1 Kurchatov Square, Moscow 123182, Russia
* Correspondence: lmkustov@mail.ru or lmk@ioc.ac.ru or lmk@mailru

Table S1. Lethal effects of Ag NPs, stabilizers, and Ag^+ ions on sea urchin embryos.

Sample	Stock solution concentration, mg/L	Exposed concentration, mg/L	Duration of exposure, h	
			Fertilized egg	Hatched blastula
Ag/STAPCG	3000/48000	0.15/2.4*	8.5	Sublethal effect
		0.3/4.8	ND[a]	2
		0.6/9.6	ND[a]	2
STAPCG	48000	2.4	8	Sublethal effect
		4.8	8	2
		10	5.5	ND[a]
		48	2	ND[a]
Ag/PHMB&SLES	500/500/5200	0.5/0.5/5.2**	8	Sublethal effect
		1/1/10.4*	5.5	2
PHMB/SLES	500/5200	1/10.4	8.5	12
		2.5/26	5.5	2
		5/52	2	ND[a]
Ag/SADG	500/2000	0.5/2**	20	Sublethal effect
		1/4*	5.5	NT[b]
SADG	2000	4	8.5	12
		10	5.5	2
		20	5.5	ND[a]
Ag/AMA	500/4000	0.5/4**	12.5	19.5
		1/8*	8.5	ND[a]
AMA	4000	8	8	Sublethal effect
		20	5.5	2
Ag/PHMB	500/500	~2.5/2.5[d]**	9	Sublethal effect
		~5/5[d]*	5.5	ND[a]
PHMB	500	5	Sublethal effect	Sublethal effect
Ag/SLES	100/1000	0.25/2.5**	Sublethal effect	Sublethal effect

		0.5/5*	NT[c]	NT[c]
SLES	1000	5	8.5	Sublethal effect
		10	5.5	2
$AgNO_3$ / Ag^+ ions	1% / dist. H_2O 0.635% Ag^+	0.0635	9.5	Sublethal effect
		0.127	5.5	Sublethal effect
		0.32	ND[a]	11
		0.635	5.5	2

[a] ND: Not determined.

[b] Not tested at HB, since SADG concentration in Ag NPs was more than SADG MEC (2 mg/L).

[c] Not tested, since SLES concentration in Ag NPs was more than SLES MEC (2.5 mg/L).

[d] It was impossible to estimate exact lethal concentration due to crystals formation in seawater at ≥0.3/0.3 mg/L.

Blue**: Ag NPs concentration was equal to MLC of the respective stabilizer.

Green*: Ag NPs concentration exceeded MEC of the respective stabilizer.
